# Deterioration of visual quality and acuity as the first sign of ceroid lipofuscinosis type 3 (CLN3), a rare neurometabolic disease

**DOI:** 10.1007/s11011-022-01148-5

**Published:** 2022-12-28

**Authors:** Joanna Karolina Purzycka-Olewiecka, Katarzyna Hetmańczyk-Sawicka, Tomasz Kmieć, Dominika Szczęśniak, Joanna Trubicka, Maciej Krawczyński, Maciej Pronicki, Agnieszka Ługowska

**Affiliations:** 1 Department of Genetics, Institute of Psychiatry and Neurology, Warsaw, Poland; 2 Department of Neurology and Epileptology, The Children’s Memorial Health Institute, Warsaw, Poland; 3 Department of Pathology, The Children’s Memorial Health Institute, Warsaw, Poland; 4 Department of Medical Genetics, Poznan University of Medical Sciences, Poznan, Poland; 5 Center for Medical Genetics GENESIS, Poznan, Poland

**Keywords:** CLN3, Batten disease, Juvenile neuronal ceroid lipofuscinosis, Vision loss, Lysosomal disease, Neurometabolic disease, Epilepsy

## Abstract

Ceroid lipofuscinosis type 3 (CLN3) is an autosomal recessive, neurodegenerative metabolic disease. Typical clinical symptoms include progressive visual loss, epilepsy of unknown etiology and dementia. Presence of lipofuscin deposits with typical pattern of ‘fingerprints’ and vacuolized lymphocytes suggest the diagnosis of CLN3. Cause of CLN3 are mutations in the *CLN3* gene, among which the most frequently found is the large deletion 1.02 kb spreading on exons 7 and 8. We present 4 patients from 2 families, in whom the deterioration of visual quality and acuity was observed as first clinical sign, when they were a few years old and it was successively accompanied by symptoms of neurologic deterioration (like generalized convulsions with consciousness impairment). In all patients the 1.02 kb deletion in the *CLN3* gene was detected in homo- or heterozygosity with other *CLN3* pathogenic variant. Ultrastructural studies revealed abnormal structures corresponding to ‘fingerprint’ profiles (FPPs) in conjunctival endothelial cells. It should be emphasized that in patients with blindness of unknown cause the diagnosis of ceroid lipofuscinosis should be considered and in older children—especially CLN3. The facility of the analysis for the presence of 1.02 kb deletion and economic costs are a solid argument for intensive use of this test in the diagnostic procedure of CLN3.

## Introduction

Lysosomal storage diseases (LSDs) are a group of inherited metabolic disorders, caused by non-functional lysosomal system in the cell. Lysosomes are small structures involved in the degradation of composed high-molecular substrates at the cellular level. As a consequence, simple small-molecule compounds can be reintroduced into the metabolic cycle. Not only inactive lysosomal hydrolases, but also non-functional lysosomal proteins of various types (including membrane proteins, transporters, activators, and others) are responsible for the storage of undegraded material.

Clinical picture of lysosomal diseases (LSDs) is very heterogeneous, but in fact the majority of them display significant neurological components ranging from ataxia to dementia. Both, central and peripheral nervous systems are affected. Thus, LSDs can also be described as neurometabolic disorders.

Among LSDs, a group of neuronal ceroid lipofuscinoses (NCLs) is distinguished, which are considered to be the most frequent cause of neurodegenerative diseases in children. Fourteen types of NCLs (CLN1 – CLN14) belong to the group, with a wide spectrum of age of onset and the rate of disease progression. Common neurological signs of all NCLs are seizures and vision loss (Schulz et al. 2013).

Ceroid lipofuscinosis type 3 (CLN3; MIM #204,200), also known as Spielmeyer–Vogt–Sjögren–Batten disease or shortly—Batten disease or juvenile neuronal ceroid lipofuscinosis (JNCL), is a rare lysosomal disorder inherited in an autosomal recessive trait (Mole and Cotman 2015). Its frequency ranges from 0.71 per 100,000 live births in West Germany to 1 per 21,000 live births in Finland (Nita et al. 2016). CLN3 is caused by mutations in the *CLN3* gene (Eiberg et al. 1989; Mitchison et al. 1997), leading to the impaired lysosomal transmembrane protein – Battenin (CLN3 protein). Currently, there are over 140 pathogenic variants detected in *CLN3* (on the basis of UniProt, ClinVar, VarSome and PubMed – Total number of variants – 661, including: Pathogenic – 142, Uncertain significance – 292, Benign – 227; accession in September 2022). The most common 1.02-kb deletion (also known as 1 kb deletion) results in a frame shift and premature termination of amino acid chain synthesis but it still allows to retain residual function of CLN3 protein, which likely explains, why this form of CLN shows later onset and less severe clinical manifestations compared to other forms of CLNs (Kitzmüller et al. 2008). The *CLN3* gene is located on chromosome 16p12.1. It contains 16 exons and encodes a deduced 438-amino acid protein named CLN3 or Battenin (The IBDC 1995; Cotman et al. 2013; Mirza et al. 2019). Diverse methods and model systems (from yeasts to human cells) shed a light onto possible roles of CLN3, which include:

- regulation of cytoskeletal/cytoskeletal-associated proteins (actin cytoskeleton and dynein motor-RILP) to tether cellular membranes,
- regulation of membrane complexes such as channels/transporters,
- modulating the function of small GTPases to effectively mediate vesicular movement and membrane dynamics (such as fission, fusion, chemotaxis/cell migration) (Cotman and Lefrancois 2021).

**Fig. 1:**
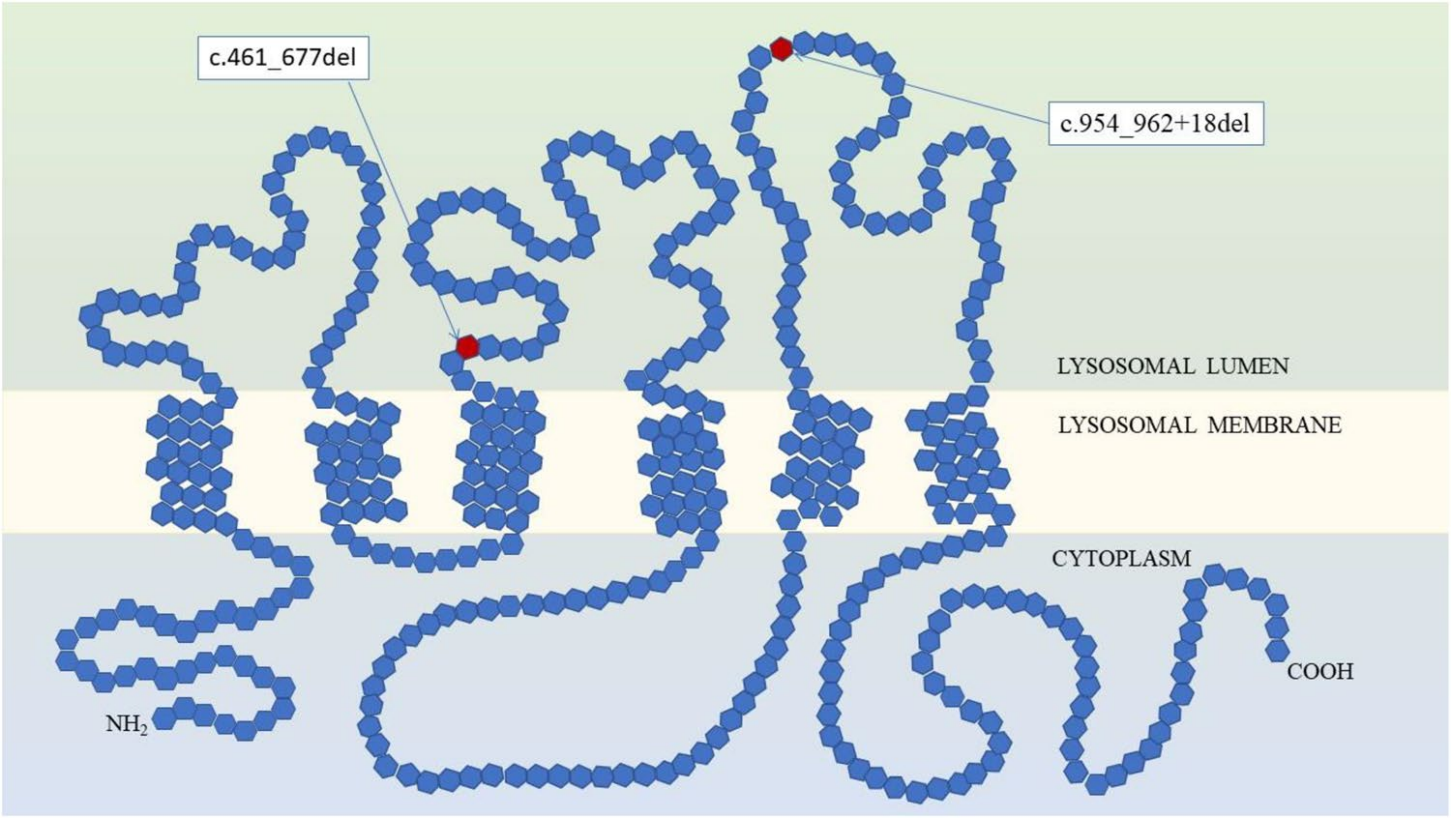
**Schematic representation of the structure of the CLN3 protein** (on the basis of Cotman and Staropoli 2012). CLN3 protein is composed of six transmembrane domains, three lysosomal luminal loops and two cytoplasmic loops. N- and C-terminus are located in the cytoplasmic interior. In red are indicated places corresponding to CLN3 pathogenic variants detected in described patients

Cotman and Staropoli suggested that CLN3 may also be involved in the incorporation of sphingolipids into lipid raft microdomains in the plasma membrane (Cotman and Staropoli 2012). Additionally, in CLN3 model cells disturbances in the sorting of the mannose 6-phosphate receptor (M6P-receptor) to the lysosome were observed, which possibly can result in the mistargeting of lysosomal hydrolases (Cotman and Staropoli 2012). Generally, the CLN3 protein may participate in anterograde and retrograde trafficking between the Golgi network, endosomes, autophagosomes, lysosomes and plasma membrane (Fig. 1). Stored material in CLN3 is mainly subunit c of the mitochondrial ATP synthase F0, which is the pore forming, membrane-spanning subunit of the mitochondrial ATP synthase complex. Accumulated compounds form mainly ‘fingerprint’ pattern (Cotman and Staropoli 2012).

The first symptoms of CLN3 occur between 4 and 10 years of life. Severe visual impairment progresses rapidly and within one to two years leads to vision loss. Epilepsy in the form of generalized tonic–clonic seizures or complex partial seizures appears usually after the age of 10 yrs. Patients survive until the age of about 30 yrs.

Typical clinical symptoms include:

- impaired vision, which usually appears between the ages of 4 and 7 years (macular degeneration, optic atrophy, and retinal degeneration)
- epilepsy of unknown etiology (may not manifest at all)
- motor dysfunction due to extrapyramidal, slight pyramidal, and cerebellar disturbances
- slowly developing mental retardation
- dementia becomes profound later in the course of the disease
- disordered speech leading to dysarthria
- presence of lipofuscin deposits with typical pattern of ‘fingerprints’ and vacuolized lymphocytes in electron microscopy (EM) suggests the diagnosis of CLN3 (Ostergaard 2016).

So far, no causal treatment for CLN3 is available. Studies performed on cell and animal models include gene therapy, immunomodulation, neuroprotection, or small-molecule therapies. Kohlschütter et al. suggest that, similarly to other lysosomal diseases and NCLs, combination of various strategies might be most effective. Moreover, to achieve satisfactory results therapeutic strategies have to target not only the brain, but also the spinal cord, the retina, and peripheral organs (Kohlschütter et al. 2019).

Our aim was to report retrospective studies in patients with visual impairment as the first type of symptoms that can easily be corroborated by carrying out a quick screening for the commonest CLN3 causal mutations. This case presentation adds supplementary evidenced medicine findings to the current knowledge on CLN3.

## Patients

### Patient 1 and Patient 2

**Fig. 2:**
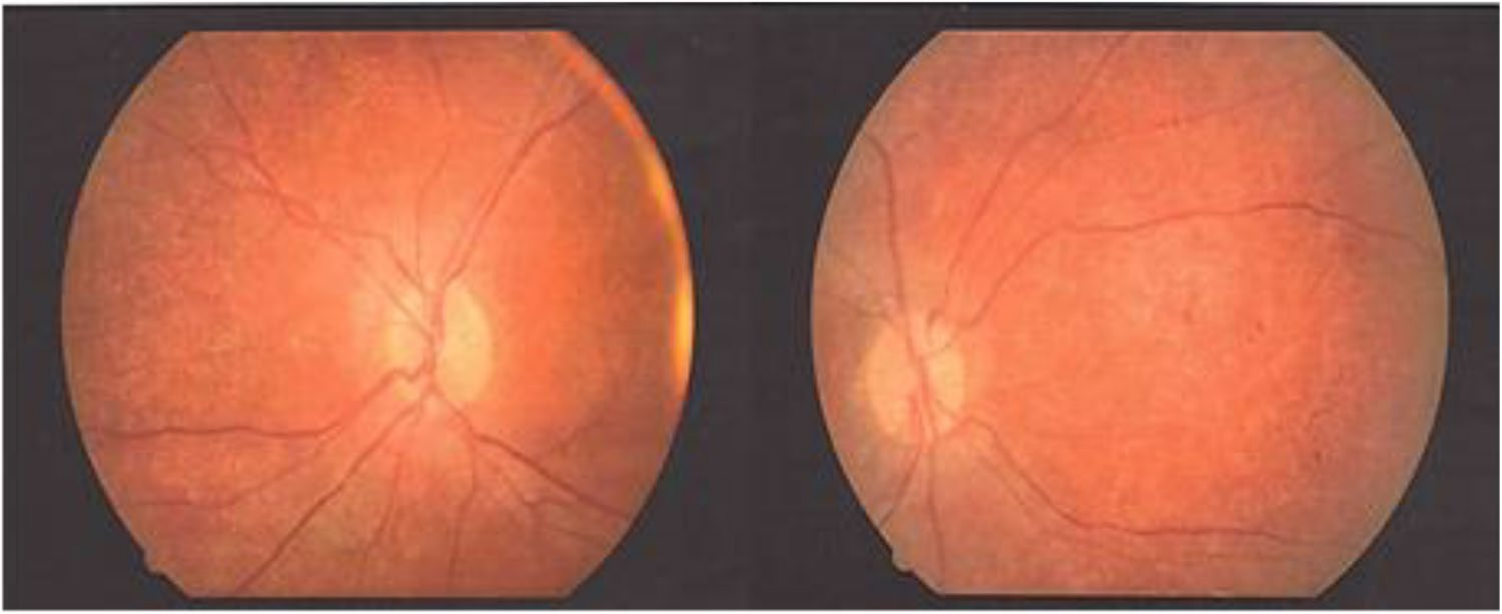
**Picture of fundus oculi in Patient no. 1**. Discs of nerve II with clear boundaries. In the peripheral parts of retina large pigment changes with the presence of ‘bone bodies’ are visible

**Patient 1** is a boy born as a healthy child to unrelated Polish parents. At the age of 14 yrs. he was under our observation at the Department of Neurology and Epileptology, The Children's Memorial Health Institute, Warsaw, Poland. First clinical signs were observed when the patient was 5 yrs. old and they included the deterioration of visual quality and acuity, accompanied by first generalized convulsions with consciousness impairment. When he was 14 yrs. old, he was still able to learn at school, but his cognition functions diminished and contact with colleagues worsened. Speech was normal. Swallowing was good and there were no choking episodes. Sleep was good. Neurological examination revealed: paraparesis spastica with Babinski sign and knees areflexia, without gait difficulties. MRI of head (age 13 yrs.) was normal. EEG (electroencephalography) (14 yrs.) was normal, EMG – NCS (electromyography and nerve conduction study) normal, ERG (electroretinogram) abnormal. Fundus oculi: discs of nerves II borders are evident, massive pigment changes on peripheral retina with ‘bone bodies’ are present, see Fig. 2. Acuity and vision – patient is able only to see fingers movement. Leber disease was excluded. Results of molecular analysis indicated the presence of homozygous 1.02 kb deletion in CLN3 gene, what confirmed CLN3 diagnosis.

**Fig. 3:**
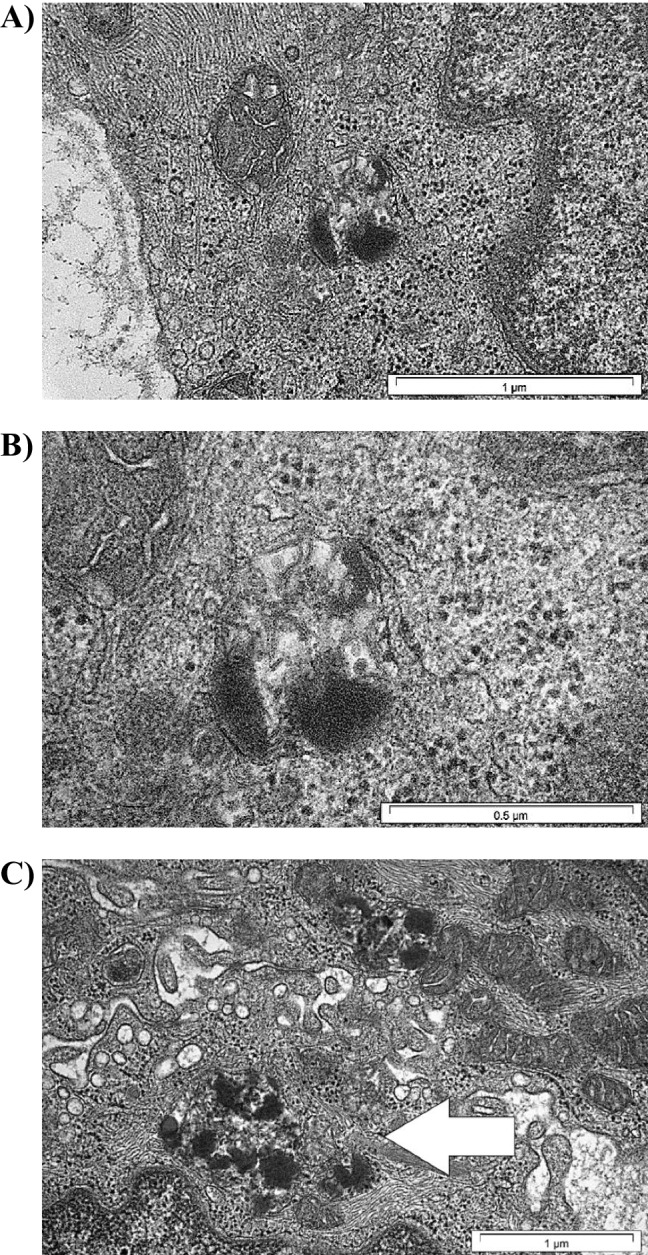
**Storage of lipofuscin in conjunctiva biopsy from Patients 2 and 4**. Transmission electron microscopic scan of conjunctiva biopsy – visible characteristic storage of lipofuscin in the form of ‘fingerprints’. A) Patient 2, 75 000x magnification; B) Patient 2, 150 000x magnification; C) Patient 4, 50 000x magnification, arrow indicates ‘fingerprints’ inclusions

**Patient 2** is a 5 years older sister of patient no. 1. She was also born healthy. Clinical picture is very similar to her brother (first symptoms also included deterioration of visual quality and acuity when she was a few years old, after which neurological symptoms joined in the same order of appearance). When she was 19 yrs. old, she stopped walking, small myoclonic jerks in facial muscles were present and the ability of swallowing has worsened. Electron microscopic analysis of conjunctiva biopsy revealed storage of lipofuscin in the form of ‘fingerprints’, see Fig. 3A and B. Biochemical analyses excluded CLN1 and CLN2: PPT1 (palmitoyl-protein thioesterase 1) activity in peripheral blood leukocytes was 54 nmol/mg protein/hr (normal range: 53 ± 18); TPP1 activity was 74 U/mg protein/hr (normal range: 54 ± 18). Additionally, Leber disease was excluded. The DNA samples from her or parents of Patients 1 and 2 were not obtained, so that we were unable to perform the molecular genetic analyses.

### Patient 3 and patient 4

At the age of 12 yrs. **Patient no. 3** and his 7-year-old sister (**Patient no. 4**) were referred to Center of Medical Genetics GENESIS in Poznan, because of clinical diagnosis of severe early-onset retinal dystrophy. At the moment of the first visit they both suffered from the ocular symptoms. The boy manifested severe nystagmus (since the age of 3 yrs.), very low visual acuity (approx. 0,01), pigmentary degeneration of the retina, atrophy of the optic nerves and extinguished ERG responses, a finding confirmed by nerve thinning in MRI and marked conduction disturbances in visual evoked potentials study (VEP). His sister manifested very low visual acuity (approx. 0,04) without nystagmus, but with extinguished ERG responses. The clinical diagnosis of Leber congenital amaurosis (LCA) was suspected. Both sibling were tested with APEX single nucleotide polymorphism microarray, covering 641 known mutations in 13 genes causative of LCA (Asper Ophthalmics, Tartu, Estonia) but no mutations were found. Patients’ 3 and 4 non-consanguineous parents were in good health. Patient 3 was born from uncomplicated pregnancy, with the Apgar score of 10/10. Since birth he displayed a physical and developmental delay.

At the age of 14 yrs., Patient no. 3 experienced his first tonic–clonic seizures. An interictal EEG showed changes in the anterior region, in the form of groups of slow waves against the background of the preserved basic activity. The diagnosis of focal epilepsy was made, and the carbamazepine treatment was initiated. MRI showed a hypointense changes in the T2 / FLAIR sequence in pale globules, otherwise without significant focal changes in the brain, brainstem and cerebellum. Neurological assessment revealed increased tension in the muscles of four limbs.

At the age of 18 yrs., Patient no. 3 was referred to the Institute of Psychiatry and Neurology due to cognitive impairment, behavioural problems, epilepsy, and bilateral optic nerve atrophy.

At the first diagnostic stage we excluded the connection of progressive blindness of the siblings with the presence of mutations in the first candidate gene *CNGB1* (based on a family study two mutations in *cis* position were identified).

In the next step, WES (whole exome sequencing) analysis allowed identification of one mutation in the *PPT1* gene (NM_001142604.1:c.274 T > C; p.(Tyr92His); CLN1) and deletion in the *CLN3* gene (NM_001042432.1: c.954_962 + 18del; p.Arg320_Tyr322del).

Both variants were rare and potentially pathogenic, but were present in only one copy (in a heterozygous state). The *CLN3* deletion c.954_962 + 18del is a known variant leading to a splice site defect. Its presence is associated with the occurrence of juvenile form of CLN3 (Kousi et al. 2012).

Reanalysis of the phenotype as part of a hospitalization in the Metabolic Unit of the The Children's Memorial Health Institute revealed the presence of discrete neurological symptoms in the siblings, which indicated a possible diagnosis of ceroid lipofuscinosis. Further investigations confirmed this diagnosis. Conjunctival biopsy has been taken from Patient 4, fixed in glutaraldehyde and processed routinely for transmission electron microscopy. Ultrastructural studies revealed abnormal structures corresponding to ‘fingerprint’ profiles (FPPs) in conjunctival endothelial cells (Fig. 3C). Additional DNA analysis for the common deletion in the *CLN3* gene (NM_001042432.1: c.461-280_677 + 382del; p. p.[Gly154Ala fs*29, Val155_Gly264del] also known as c.461_677del or 1.02 kb deletion) showed the presence of this variant in Patient 3 in a heterozygous state. Moreover, it was found that the activity of PPT1 (which deficient activity is responsible for CLN1 – late infantile neuronal ceroid lipofuscinosis, LINCL) in peripheral blood leukocytes was found to be within the heterozygous state: 17 nmol/mg protein/hr (reference range: 53 + 18.6 nmol/mg protein/hr). This result was in agreement with the mutation carrier state in *PPT1* gene (detected by WES). Finally, the diagnosis of ceroid lipofuscinosis type 3 was made on the basis of detection of two pathogenic variants in a heterozygous state in the *CLN3* gene, see Table 1.

**Table 1:** Family segregation analysis of pathogenic variants detected in Patient 3 and 4 responsible for CLN1 and CLN3

	Allele 1	Allele 2
Mother	CLN3: c.461_677del CLN1: c.274 T > C	CLN3: - CLN1: -
Father	CLN3: c.954_962 + 18del	CLN3: -
Patient 3	CLN3: c.461_677del CLN1: c.274 T > C	CLN3: c.954_962 + 18del
Patient 4	CLN3: c.461_677del CLN1: c.274 T > C	CLN3: c.954_962 + 18del

Patient’s 3 sister (e.g. Patient 4) is also a compound heterozygote for both identified deletions in the *CLN3* gene. Described sibling inherited deletion c.954_962 + 18del from their father, while the big deletion c.461_677del was of maternal origin.

Both, Patient 3 and his sister were carriers for the p.(Tyr92His) mutation in the *PPT1* gene and it was also detected in the siblings' mother. We assume that additionally detected in this family the novel mutation p.(Tyr92His) in the *PPT1* gene is accidental and is not related to the occurrence of clinical symptoms.

## Discussion

Disorders of eye structure and function are a hallmark of numerous inherited metabolic diseases, including neuronal ceroid lipofuscinoses (NCLs). While in many metabolic diseases these ophtalmic involvement include: 1) eye movement disorders (oculogyric crisis, ophthalmoplegia, ptosis); 2) corneal pathology (keratopathy, corneal clouding/opacification); 3) lens pathology (ectopia lentis, cataract); 4) glaucoma; and 5) retina (retinitis pigmentosa, cherry red spot, optic atrophy), in NCLs they present mainly as retinitis pigmentosa and progressive optic atrophy, leading to visual loss (Davison 2020).

In CLN3 patients described here, loss of visual quality and acuity were one of the first clinical symptoms, which appeared during the early childhood. In juvenile NCLs patients, additionally nyctalopia, nystagmus, photophobia, and loss of peripheral and colour vision, can also be often the presenting symptoms (Ouseph et al. 2016). These pathologic changes are most probably caused by the diffuse retinal pigment epithelium (RPE) atrophy of the macula, which appeared in over 60% of juvenile patients with NCL (Hainsworth et al. 2009), but the pathomechanism underlying vision loss in CLN3 individuals remains still unexplained.

The retina, which is a photosensitive nerve tissue, is a functional unit of the central nervous system (CNS) that converts a light signal into a nerve impulse and is physically connected to the brain via axons of the optic nerve (Marchesi et al. 2021). As a part of CNS, the pathologically changed eye reflects neurodegeneration present in the CNS and sometimes visual manifestations precede central symptoms (Marchesi et al. 2021).

Disrupted metabolism and stored material in CLN3 cells lead to neurodegeneration manifested by cerebral and cerebellar atrophy and significant volume reduction in affected areas of the brain, visible on magnetic resonance (MR) images (Ouseph et al. 2016). Cellular pathologic changes result in progressive loss of function of neurons and are resembling degeneration processes taking place in other neurodegenerative CNS pathologies like Alzheimer disease and Parkinson disease, glaucoma, amyotrophic lateral sclerosis (ALS), age-related macular degeneration (AMD), diabetic retinopathy (DR), sclerosis multiplex (SM), retinitis pigmentosa (RP), Huntington’s disease (HD) (Marchesi et al. 2021) and neurodegeneration with brain iron accumulation (NBIA; former Hallervorden-Spatz disease).

Storage of subunit c of the mitochondrial ATP synthase complex in CLN3 cells and tissues results in stimulation of autoimmune response, extensive neurodegeneration, and gliosis (Bozorg et al. 2009). Neuroinflammatory processes triggered in CLN3 cells can be responsible for a loss of brain matter, impairment of the retina and injury to the optic nerve and/or retinal ganglion cells (Gupta et al. 2016). This pathomechanism is observed also in other neurodegenerative diseases like Alzheimer disease and glaucoma, in which excitation of microglial cells is associated with injury and degeneration of neurons (Marchesi et al. 2021).

Although both pairs of siblings described by us showed the same symptoms, the centers to which they were admitted took different first diagnostic steps. It should be emphasized that due to the unclear etiology of eye lesions and progressive loss of vision, the availability of the WES method would provide a much faster solution, and patients would avoid the so-called diagnostic odyssey. Supplying patients with proper diagnostics, even in the absence of an available treatment is of great importance for them and their families. Next-generation molecular tests give this possibility and are becoming increasingly common.

Interestingly, on the basis of published findings, Mirza et al. (2019) summarized that the most frequent mutation c.461_677del maps within the second luminal loop of CLN3 amino acid chain, which sequence is the most highly conserved among species, and which spreads into the lysosomal lumen. The second CLN3 mutation identified in patients 3 and 4, namely c.954_962 + 18del, maps to the third luminal loop stretching between the fifth and sixth transmembrane helices (Mirza et al. 2019). These two luminal loops must be of critical importance for the interaction and function of CLN3 with other proteins due to the fact that majority of missense mutations accumulates in the DNA region coding for this part of CLN3 protein molecule (Mirza et al. 2019; Cotman and Staropoli 2012).

It is also important to consider ceroid lipofuscinosis in patients with blindness of unknown cause and in older children—especially CLN3. The facility of the analysis for the presence of 1.02 kb deletion and low economic costs are a solid argument for intensive use of this test in the diagnostic procedure of CLN3.

## Data Availability

The datasets analysed during the current study are not publicly available due to protection of personal data of described patients but are available from the corresponding author on reasonable request.
